# Reproductive Trade-Offs in *Culex pipiens*: Effects of CYV Infection and Delayed Mating

**DOI:** 10.3390/insects16030252

**Published:** 2025-03-01

**Authors:** Mareike Heinig-Hartberger, Fanny Hellhammer, Stefanie C. Becker

**Affiliations:** 1 Institute for Parasitology, University of Veterinary Medicine Hannover, Buenteweg 17, 30559 Hannover, Germany; mareike.heinig@tiho-hannover.de (M.H.-H.); fanny.hellhammer@tiho-hannover.de (F.H.); 2 Research Center for Emerging Infections and Zoonoses, University of Veterinary Medicine Hannover, Buenteweg 17, 30559 Hannover, Germany

**Keywords:** insect-specific virus, CYV, fecundity, reproductive success, *Culex pipiens*

## Abstract

Arbovirus control strategies often target vector reproductive dynamics, with insect-specific viruses (ISVs) like Culex Y virus (CYV) offering potential as eco-friendly approaches by influencing mosquito reproduction without affecting humans or animals. This study investigated the interplay between autogeny, anautogeny, nutrient availability, and viral infection and their effect on reproductive success in *Culex pipiens* biotype *molestus* and *Culex pipiens quinquefasciatus*. CYV infection had a minimal impact on mosquito reproductive parameters, even after a five-day incubation period. Autogeny enabled *Cx. pipiens* biotype *molestus* to produce eggs without blood meals, yet older females (3–5 days post-emergence) showed reduced reproductive success unrelated to nutrient deficiency, as blood-feeding did not restore their egg production. These findings demonstrate that age affects reproductive success in *Cx. pipiens* biotype *molestus* but not in *Cx. pipiens quinquefasciatus* and suggest that CYV has negligible direct effects on mosquito reproduction. This work enhances our understanding of ISV biology and vector ecology, supporting the development of innovative, sustainable arbovirus-control strategies.

## Simple Summary

Mosquitoes are important carriers of viruses that cause diseases like West Nile virus and Zika virus, and controlling their reproduction can help reduce the spread of these diseases. One potential approach to controlling mosquito populations is using viruses that specifically affect mosquitoes, known as insect-specific viruses. In this study, we examined the impact of the Culex Y virus on the reproduction of two mosquito species: *Culex pipiens* biotype *molestus* and *Culex pipiens quinquefasciatus*. We also compared these mosquitoes to better understand how different species respond to the virus. Our results showed that the virus did not have a major impact on mosquito reproduction, although the age of the mosquitoes did affect their ability to produce eggs as well as their general oviposition behavior. Older *Cx. pipiens* biotype *molestus* mosquitoes laid fewer eggs, even when they were given a blood meal. These findings are important for understanding influential factors for mosquito reproduction and can help improve strategies for controlling mosquito populations without harming other animals or humans.

## 1. Introduction

Vector-borne diseases pose a major threat to public health due to their significant impact and widespread consequences. Of particular concern are viral pathogens that are transmitted to humans by mosquitoes (arboviruses), which have shown a troublesome increase in incidence and geographical range in recent years [1]. An important group in this context is the *Culex pipiens* complex, which is found worldwide and includes several species that transmit arboviruses that affect both human and animal health. Notable examples include *Orthoflavivirus nilense* (West Nile virus; WNV) [2], *Phlebovirus riftense* (Rift Valley fever virus; RVFV) [3], *Orthoflavivirus lousense* (St. Louis encephalitis virus; SLEV) [4], and *Alphavirus sindbis* (Sindbis virus; SINV) [5]. Recent outbreaks of WNV in Europe [6] and the ability of the *Cx. pipiens* complex to form hybrids, thereby expanding its distribution and vector range [7], highlight the limitations of current measures [8]. Given the slow progress of vaccine development for many arboviruses, improving vector control strategies remains a critical priority. Accordingly, various new strategies have been researched and described in recent years to better predict or even prevent outbreaks [9,10].

A cutting-edge strategy involves the utilization of biological agents derived from the vector’s microbiome to enhance control of that vector. The microbiome of a vector, comprising microorganisms such as bacteria, viruses, protozoans, and fungi, wields a substantial influence on the phenotype of its host, encompassing the vector’s competence and reproductive biology. In particular, insect-specific viruses (ISVs), discovered through the extensive monitoring of arboviruses [11], could be an interesting option for modifying vector competence or controlling vector populations. However, the impact of these viruses on arthropod hosts, particularly their effect on arthropod reproduction, remains poorly understood. It is imperative to understand these interactions, as they may play a crucial role in shaping vector population dynamics and potentially influence the transmission of arboviruses.

In this study, we examined the effect of CYV, a member of the *Birnaviridae* family which was first detected in *Cx. pipiens* mosquitoes in 2010 [12], on mosquito reproduction. CYV has been found to replicate in various mosquito species, including *Aedes albopictus* and *Cx. pipiens* biotype *molestus*. A previous study found the replication of CYV in ovarian tissues of *Cx. pipiens* biotype *molestus* mosquitoes but no significant differences in their egg-laying, larval hatching, pupation, or adult emergence when they were mated shortly after infection. However, the full scope of its effects on mosquito fecundity and fertility remains to be elucidated [13,14]. The particularly extended incubation periods of CYV infection, leading to high CYV titers in reproductive organs, could lead to significant changes in reproductive traits.

Understanding the mating biology of the *Cx. pipiens* complex is crucial due to its role as a vector. Most research, however, emphasizes *Aedes aegypti* [15], *Ae. albopictus* [16,17], and the malaria-transmitting *Anopheles* species [11,18], focusing on factors such as swarm dynamics, mating success, and mating age as significant influences on mosquito reproduction [19]. In contrast, studies of *Cx. pipiens* complex mosquitoes are scarce and often focus on male mating and swarming behavior [20,21,22,23,24]. The *Cx. pipiens* complex, including *Cx. pipiens* biotype *molestus* and *Cx. pipiens quinquefasciatus*, has adapted to the human population by utilizing artificial habitats for oviposition. *Culex pipiens* biotype *molestus* females are stenogamous (capable of mating in confined spaces), mammophilic (preferring mammalian blood), and autogenous (able to lay their first egg batch without a blood meal). They remain active year-round, breeding in warm, nutrient-rich underground environments [25,26]. In contrast, *Cx. pipiens quinquefasciatus*, which is prevalent in tropical urban areas, is also stenogamous but is anautogenous, requiring a blood meal for oviposition, and lacks the ability to enter diapause, a state of developmental arrest that allows mosquitoes to survive unfavorable environmental conditions. This distinguishes it from the “northern house mosquito” (*Cx. pipiens*), which survives winters through diapause, stress tolerance, and lipid storage [27].

This study aims to elucidate the mechanisms underlying insect reproductive biology by integrating research on CYV infection in *Cx. pipiens* biotype *molestus* (autogenous species) and *Cx. pipiens quinquefasciatus* (anautogenous species). The findings could potentially contribute to the development of innovative approaches aimed at disrupting mosquito populations and reducing the burden of vector-borne diseases.

## 2. Materials and Methods

### 2.1. Mosquito Rearing

Laboratory strains of two biotypes of the *Cx. pipiens* complex were used in this study. The *Cx. pipiens* biotype *molestus* strain (Forskal, 1775), abbreviated in the following as “*Cm*”, is a hybrid strain of two strains from northern Germany which was established in Hannover in 2023. The colony was maintained in climate chambers at 26 °C (±1 °C) and a relative humidity of 45–75%, with a 16:8 (light:dark) photoperiod with 1 h twilight periods at dusk and dawn. The subspecies *Cx. pipiens quinquefasiatus* strain (Say, 1823), abbreviated as “*Cq*” in the following, was provided by the Bernhard Nocht Institute, Hamburg, Germany, and established in Hannover in 2020. The *Cq* colony was maintained at 28 °C (±1 °C) and a relative humidity of 60–75%, with a 16:8 (light:dark) photoperiod with 1 h twilight periods at dusk and dawn. The larvae of both species were reared in plastic basins filled with stale tap water and fed with TetraPleco fish food tablets (Tetra Werke, Melle, Germany). After emergence, adult mosquitoes were housed in rearing cages (BugDorm-1, 30 cm × 30 cm × 30 cm; Bioquip, Compton, CA, USA) and fed *ad libitum* with 8% fructose solution supplemented with 0.5 g/L of 4-aminobenzoic acid (PABA). Once a week, adult mosquitoes were fed with animal blood.

### 2.2. Separation and Rearing of Virgin Females

**Figure 1:**
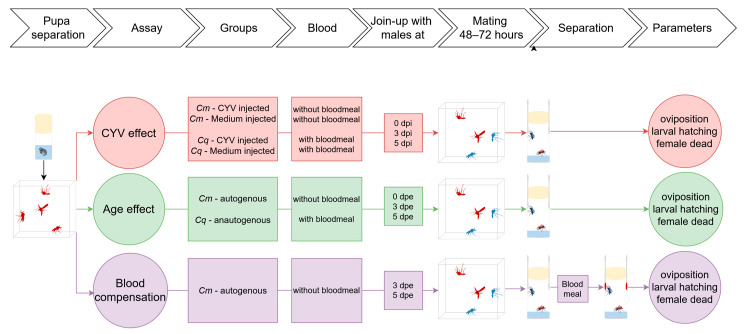
Schematic representation of the present study. The present study is comprised of multiple experiments. The red section of the schematic delineates the investigation of the effects of CYV infection on the reproduction of *Cq* and *Cm*. The green section of the study examines reproduction in *Cq* and *Cm* females as a function of age, without injections. The purple section focuses on an additional study for *Cm*, where the provision of a blood meal is highlighted in the diagram to compensate for the lower reproductive success after the autogenous phase. The measurement of laying performance, larval hatch rate, and female mortality rate constitutes a fundamental aspect of all trials, as these parameters collectively determine reproductive success.

To generate virgin female mosquitoes for all experiments (Figure 1), the pupae were separated individually in plastic vials and the emerged mosquitoes were divided into different cages according to sex. All experiments were carried out in rearing cages (BugDorm-1) and in plastic vials in a climate chamber at 26 °C (±1 °C) and a relative humidity of 45–75%, with a photoperiod of 16:8 (light:dark) with a 1 h twilight period at dusk and dawn.

### 2.3. Effect of CYV Infection on Mosquitoes’ Reproductive Success

Mosquitoes were infected with CYV derived from the supernatant of persistently infected C6/36 cells, as described in Franzke et al. [28], by microinjection using the Nanoject III auto-nanoliter injector (Drummond Scientific Company, Broomall, PA, USA) at an injection rate of 23 nL/s. The experiments were conducted in at least three individual replicates and the successful infection of the animals was checked for 10% of the injected individuals in this study. The injected females were examined either at the end of the experiment (max. 16 days after join-up with males) or when larvae hatched from laid egg-rafts. Females were individually homogenized with 500 µL Schneider’s Drosophila medium (PAN-Biotech, Aidenbach, Germany) and a 3 mm steel bead in a TissueLyser II (Qiagen, Hilden, Germany) for 30 s at 30 hertz. Viral RNA was extracted using the QiAamp viral RNA mini kit (Qiagen, Hilden, Germany) according to the manufacturer’s instructions. RT-qPCR was performed using the Luna Probe One-Step RT-qPCR Kit (NEB, Ipswitch, MA, USA), and viral RNA was quantified with a CYV standard as previously described [13,14]. Since the detection limit of the CYV standard measurement is 1 × 10⁴ copy numbers, corresponding to Ct values between 29 and 33, samples with Ct values above 33 did not meet this threshold and were therefore considered “negative”. The successful mating of each insect was not explicitly observed in all experiments. However, due to the delayed join-up of the sexes, the possible outcomes of the experiment were either that no mating occurred or that mating was delayed. For the oviposition assay, CO_2_-anesthetized virgin *Cm* and *Cq* females were injected from one to three days after emergence with either 46 nL of the CYV isolate comprising on average 5.14 × 10^4^ RNA copy numbers or 46 nL of Schneider’s Drosophila medium (control group). To analyze the effect of viral replication in reproductive tissues on oviposition, females were either mated immediately (dpi) or kept separated by sex for 3 or 5 days (3 dpi or 5 dpi) before being mated with males at a 1:1 ratio. For *Cx. pipiens* biotype *molestus* (*Cm*), 45 females per group were tested. For *Cx. pipiens quinquefasciatus* (*Cq*), 44, 70, and 48 females were injected with CYV at 0, 3, and 5 dpi, respectively, while 45, 53, and 80 females were injected with a medium solution as controls. Each treatment and time point included at least three independent replicates. The higher mortality of *Cq* after injection resulted in variable sample sizes and, for the 5 dpi group, individuals from two replicates were analyzed.

During the 48–72 h mating period, *Cm* mosquitoes were fed an 8% fructose solution, while *Cq* mosquitoes received a blood–fructose water mixture (3:1), with no oviposition sites provided. After mating, females were placed in individual vials containing stagnant water for oviposition, with males added to form pairs. *Cq* females that did not feed on blood during mating continued blood–fructose feeding until ingestion was confirmed. Observations lasted up to 16 days, ending earlier when the females died or when the larvae hatched, which defined the vial as complete. Dead males were not replaced. Observation was extended if eggs were laid near the experiment’s end to allow for larval hatching. Three parameters were assessed: oviposition rate (number of egg rafts laid), hatch rate (larvae hatched from egg rafts), and female mortality. Reproductive success was calculated as the ratio of females at the experiment’s start to egg rafts that produced larvae.

### 2.4. Effect of Delayed Mating on Mosquitoes’ Reproductive Success

Experiments were conducted with *Cm* and *Cq* females to investigate whether age prior to mating influences oviposition success, thereby examining a potential mating-time-dependent effect on reproductive outcomes. To investigate this, virgin female mosquitoes were maintained on an 8% fructose solution as their sole food source and were not provided with an oviposition site until mating. Mating occurred on day 0, day 3, and day 5 post-emergence (dpe) due to transferring of the females into a new cage with males at a ratio of 1:1. The mating period lasted 48–72 h in cages, in which *Cm* mosquitoes were provided with an 8% fructose solution and *Cq* mosquitoes received animal blood and fructose solution. Neither group was given an oviposition site during the mating period. After mating, the females were transferred to plastic vials along with up to two males. The primary parameters assessed in this experiment included oviposition rate, larval hatching rate, and female mortality. For *Cm* mosquitoes, a total of 45 females were tested per time point across at least three individual replicates. For *Cq* mosquitoes, 46 females were tested in the 0 dpe and 3 dpe groups, while 45 females were evaluated in the 5 dpe group.

### 2.5. Effect of Blood Compensation After Delayed Mating on Autogenous Culex pipiens Biotype Molestus Mosquitoes

An additional experiment was conducted to investigate whether a blood meal could mitigate the effects of delayed mating in females kept virgin for three or five days. This aimed to assess whether a blood meal could compensate for the observed reduction in the number of egg rafts and hatched larvae in the autogenous *Cm* biotype. As in the previous experiment, the setup for assay 2.4, to investigate the effect of age on fecundity, began by maintaining females as virgins for three and five days post-emergence (dpe) before introducing them into cages with males. After a mating period of 48–72 h, they were individually transferred to plastic vials. Then, the primary parameters of the oviposition rate and the larval hatching rate from the egg rafts were assessed. In contrast to assay 2.4, the experimental procedure was modified as follows: The observation period was divided into two phases. The first phase, termed the *autogenous period*, represented the first seven days after mating, during which the females had no access to blood. After this *autogenous period*, females that had not yet laid eggs were provided with a blood–fructose water mixture using cotton buds, marking the start of the *post-blood feeding period*. During this period, the number of females feeding on blood, the time point of female mortality, the general oviposition, and the larval hatch were monitored over an additional eight days. A total of 50 females from the 3 dpe group and 56 females from the 5 dpe group were tested in at least three individual replicates.

### 2.6. Protein Measurement

To quantify the amount of protein in virgin autogenous (*Cm*) and anautogenous (*Cq*) females at 0 dpe, 3 dpe, and 5 dpe, the females were separated as described in 2.4. The females were then frozen at −20 °C on the designated days for subsequent analysis. For each sampling day and species, five females were analyzed per pool, with two separate pools being analyzed for each group. All samples were then homogenized in 500 µL Tris/EDTA buffer with a 3 mm steel bead for 30 s at 30 Hz in a TissueLyser II (Qiagen, Hilden, Germany). The homogenized samples were then supplemented with an additional 450 µL of Tris/EDTA buffer and 50 µL of 20% SDS, for a final SDS concentration of 1% per pool, and incubated for 30 min at room temperature on an orbital plate. For protein measurement, the Pierce BCA Protein Assay Kit (Thermo Scientific, Waltham, MA, USA) was used according to the manufacturer’s manual for the microplate procedure.

### 2.7. Statistical Analyses

All statistical analyses were performed using GraphPad Prism 9 (GraphPad Software, San Diego, CA, USA). Normality of data was assessed using the Shapiro–Wilk test. Bonferroni corrected post hoc tests were performed when significance was detected in parametric data. Parametric data were analyzed using a one-way analysis of variance (ANOVA) or a two-way ANOVA. Non-parametric data were analyzed using the Kruskal–Wallis test, with post hoc analyses adjusted with the Benjamini–Krieger–Yekutieli correction to control for false discovery rate. Survival analyses were performed using the Kaplan–Meier survival analysis, with statistical differences assessed using the log-rank test. Categorical data were analyzed using Fisher’s exact test, with odds ratios (ORs) and 95% confidence intervals (CI) calculated to assess the strength of associations. Statistical significance was set at *p* < 0.05.

## 3. Results

### 3.1. Effect of CYV Infection on Mosquitoes’ Reproductive Success

To analyze whether CYV injection affects the reproduction in *Cq* and *Cm* mosquitoes, different incubation periods for the virus in vivo were selected before the females were allowed to mate. This approach was taken in light of findings indicating that substances from male accessory glands can influence the physiology and oogenesis of females (reviewed in [29]). Previous studies have demonstrated an increase in the CYV viral RNA copy numbers in the reproductive organs starting at 3 dpi, reaching a peak at 7 dpi in *Cm* mosquitoes [13], while, for *Cq* mosquitoes , the peak occurred earlier, at 3 dpi. To ensure ongoing exponential replication in our females—which could trigger a response in the mosquito that is relevant to reproduction—we selected day 5 for the *Cm* mosquitoes, as this timing allowed for high viral loads while still permitting continued viral replication. Different parameters have been considered for this analysis, comprising the oviposition rate of females, the larval hatch rate per egg raft laid, the female mortality, the blood meal uptake rate (only for the anautogenous *Cq* mosquitoes), and the overall reproductive success for the medium- and CYV-injected groups at 0 dpi, 3 dpi, and 5 dpi (Table 1, Supplementary Table S1). The infection rate of the females tested for CYV was 83.33% for the *Cq* mosquitoes and 75% for the *Cm* mosquitoes (Supplementary Table S2). 

**Figure 2:**
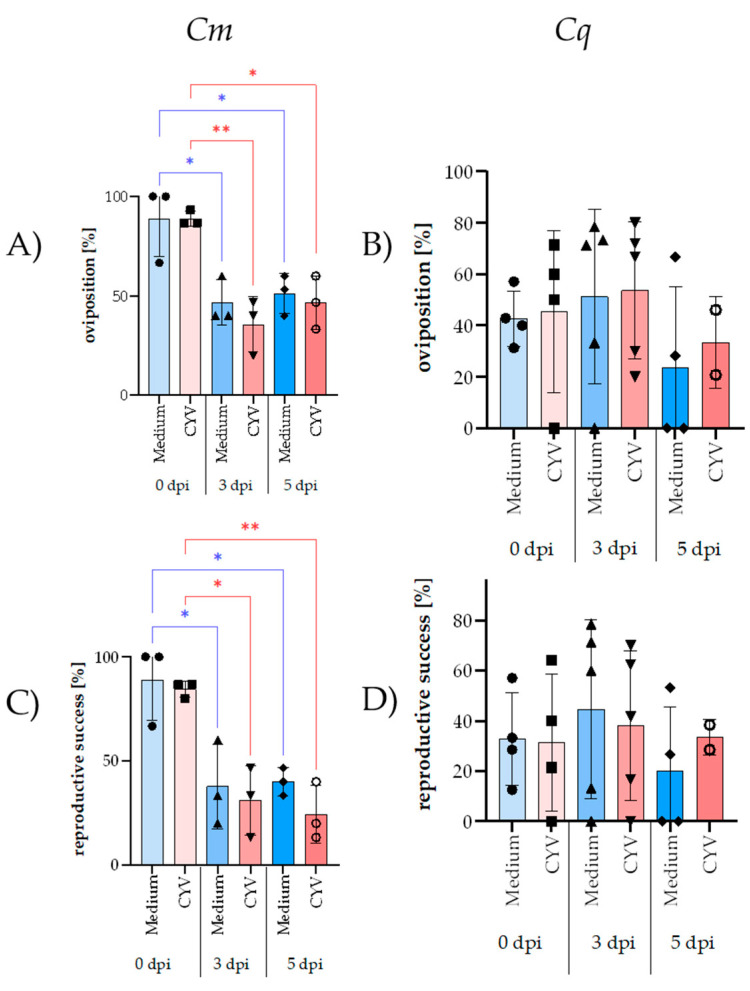
A direct comparison of the oviposition rate and reproductive success of *Cq* and *Cm* females injected with CYV or medium and mated at different time points after injection. (**A**) Oviposition rate of *Cm* females, with significant differences indicated by asterisks: medium 0 dpi vs. 3 dpi (*p* = 0.0196) and 0 dpi vs. 5 dpi (*p* = 0.0410); CYV 0 dpi vs. 3 dpi (*p* = 0.0035) and 0 dpi vs. 5 dpi (*p* = 0.0196). (**B**) Oviposition rate of *Cq*, showing no significant differences between groups. (**C**) Reproductive success of *Cm* females, with significant differences indicated by asterisks: medium 0 dpi vs. 3 dpi (*p* = 0.0275) and 0 dpi vs. 5 dpi (*p* = 0.0368); CYV 0 dpi vs. 3 dpi (*p* = 0.0206) and 0 dpi vs. 5 dpi (*p* = 0.0089). (**D**) Reproductive success of *Cq* females, with no significant differences between groups. In the figure, blue represents females injected with medium, while red represents those injected with CYV. The color gradient from light to dark indicates the time points: 0 dpi (lightest shading), 3 dpi (medium shading), and 5 dpi (darkest shading). Error bars represent the standard deviation (SD) calculated from the percentages of at least two independent biological replicates. Asterisks denote significant *p*-values (* *p* < 0.05; ** *p* < 0.01).

For the *Cm* mosquitoes, the oviposition rate in the CYV-treated groups averaged 63.5%, compared to 62.3% in the medium-treated group, with no significant difference (*p* > 0.05; *t*-test) being observed regardless of the time point (Supplementary Table S3). The overall reproductive success, measured by the oviposition and hatching rates, was only slightly reduced in the CYV group (55.6%) due to a lower mean larval hatch rate (86.4%) compared to the medium-treated group, which had higher reproductive success (57.8%) and a greater average larval hatch rate (91.3%) (*p* > 0.05; *t*-test). Mortality was not observed in the medium-treated groups, while a slight but not significantly increased mortality was observed in the CYV-treated groups. A two-way ANOVA revealed significant differences in oviposition rates and overall reproductive success within the treatment groups (Figure 2A,C). The medium group, mated at 0 dpi, had significantly higher oviposition rates and reproductive success than those mated at 3 dpi (*p* = 0.0196 and *p* = 0.0275, respectively) and 5 dpi (*p* = 0.0410 and *p* = 0.0368, respectively), although differences between the 3 dpi and 5 dpi groups were not statistically significant. The same pattern could be detected in the CYV-injected groups, which had a significantly higher oviposition rate at 0 dpi compared to that at 3 dpi (*p* = 0.0035) and 5 dpi (*p* = 0.0196), and a higher overall reproductive success rate at 0 dpi compared to that at 3 dpi (*p* = 0.0206) and 5 dpi (*p* = 0.0089). The larval hatch rate, however, was not significantly different between the different dpis.

As with the *Cm* mosquitoes, no CYV-specific effects on oviposition or larval hatching were observed in the *Cq* mosquitoes. However, delayed mating had the opposite effect in the *Cq* mosquitoes, resulting in an increase in the average oviposition rates from 40% at 0 dpi to 88.8% at 5 dpi in the medium-treated group and from 52.3% to 85.4% in the CYV-treated group. The larval hatch rates were high for both treatments, averaging 84.1% in the medium-treated group and 79.9% in the CYV-treated group. The female mortality remained consistent across all groups, with the medium-treated group showing a slightly lower but statistically non-significant average mortality rate of 18.6% compared to the 20.7% observed for the CYV-treated group. CYV infection did not affect the blood meal uptake, with an average of 98.6% blood-fed females being observed in the CYV group and 99.6% in the medium-treated group. The mean reproductive success was 54.9% for both the CYV-treated and medium-treated groups. Statistical analyses using two-way ANOVA (*p* > 0.05) revealed no significant differences in the oviposition rate (Figure 2B), reproductive success (Figure 2D), female mortality, or larval hatch rate between the different mating times or treatments.

### 3.2. Effect of Delayed Mating on Mosquito Reproductive Success

**Figure 3:**
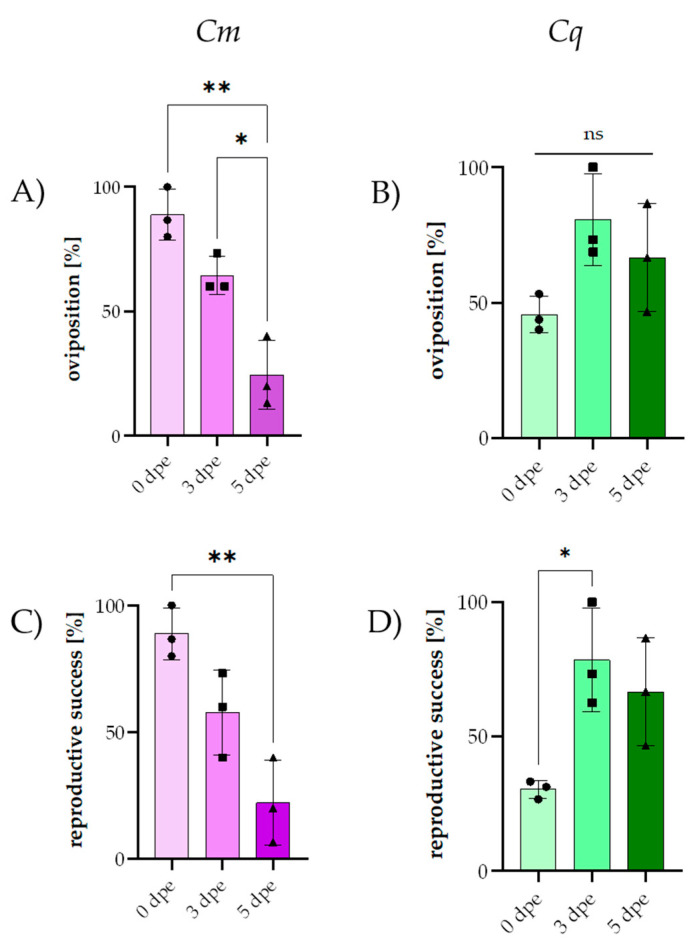
A direct comparison of the oviposition rate and reproductive success of *Cq* and *Cm* females at 0, 3, and 5 days post-emergence (dpe). (**A**) Oviposition rate of *Cm*, with significant differences indicated by asterisks: 0 dpe vs. 5 dpe (*p* = 0.001) and 3 dpe vs. 5 dpe (*p* = 0.0123). (**B**) Oviposition rate of *Cq*, showing no significant differences between groups (ns). (**C**) Reproductive success of *Cm*, with significant differences indicated by asterisks: 0 dpe vs. 5 dpe (*p* = 0.0046). (**D**) Reproductive success of *Cq*, with significant differences indicated by asterisks: 0 dpe vs. 3 dpe (*p* = 0.0321). In the figure, purple represents *Cm* females, while green represents *Cq* females. The color gradient from light to dark indicates the time points: 0 dpe (lightest shading), 3 dpe (medium shading), and 5 dpe (darkest shading). Error bars represent the standard deviation (SD) calculated from the percentages of three independent biological replicates. Asterisks denote significant *p*-values (ns *p* > 0.05; * *p* < 0.05; ** *p* < 0.01).

Given the significantly decreased reproduction in the 3- and 5-day joint groups of *Cm* mosquitoes, we analyzed the reproductive success of *Cm* and *Cq* mosquitoes based on their age at mating. For the *Cq* mosquitoes, an average of 91.2% females took a blood meal across all groups, and only these females were included in the calculation. Overall, the average female mortality in the *Cq* mosquitoes was 2.9%, with the highest recorded value being at 6.5% in the 0 days post-emergence (dpe) group. The average oviposition rate of the *Cq* mosquitoes across all experimental groups was 68.6%, with the lowest rate of 46.7% being at 0 dpe and highest of 84.1% being at 3 dpe. The larval hatch rates were also the lowest in the 0 dpe group (66.7%) but reached 100% in the 5 dpe group. The reproductive success was the lowest in the 0 dpe group at 31.1%, increasing to 81.1% in the 3 dpe group. Generally, the average reproductive success across all groups was slightly higher for the *Cq* mosquitoes with 62.6% as compared to 56.3% for the *Cm* mosquitoes (Table 2, Supplementary Table S4). As observed in the CYV infection experiment (Table 1, Figure 1), there was a notable difference between the two species regarding the timing of peak reproductive success. For the *Cm* mosquitoes, the highest oviposition rate and reproductive success (Figure 3C) occurred at 0 dpe (>88%), with a significant decline by 5 dpe (<25%) (*p* = 0.0046, ordinary one-way ANOVA). Additionally, significant differences in the oviposition rate were found between 5 dpe and both 0 dpe (*p* = 0.001) and 3 dpe (*p* = 0.0123) for (Figure 3A), as determined by ordinary one-way ANOVA (Table 2). For the *Cq* mosquitoes, no significant differences in oviposition rates (Figure 3B) were found for all time points (simple ANOVA *p* > 0.05). However, the reproductive success (Figure 3D) was significantly higher in the 3 dpe group than in the 0 dpe group (one-way ANOVA *p* = 0.0321).

**Figure 4:**
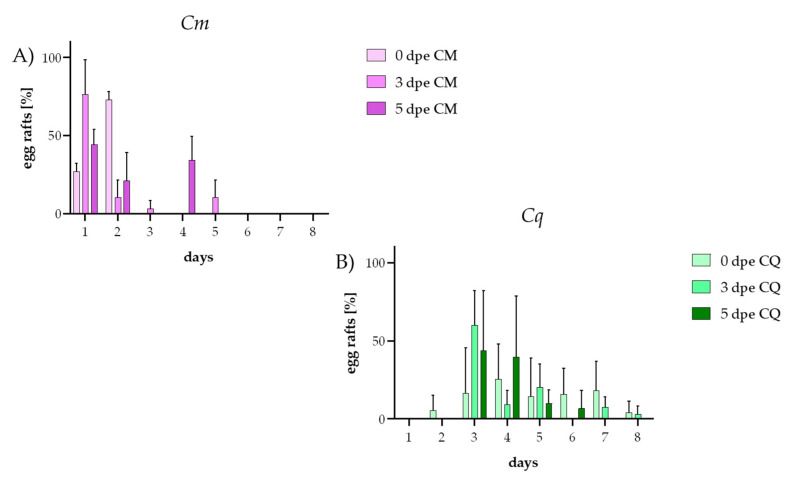
Oviposition time points after separation into oviposition tubes of *Cq* and *Cm* females at 0, 3, and 5 dpe. (**A**) Oviposition time points of *Cm*, (**B**) oviposition time points of *Cq*. The color gradient from light to dark represents 0 dpi (lightest shading), 3 dpi (medium shading), and 5 dpi (darkest shading). Error bars indicate the standard deviation (SD).

Using two-way ANOVA, we further analyzed the differences in the temporal patterns of oviposition for *Cm* and *Cq*. The onset and temporal distribution of oviposition differed significantly between the *Cm* and *Cq* mosquitoes. In the case of *Cm* (Figure 4A), oviposition onset decreased significantly over time as described above. Concerning the egg-laying pattern, of the 40 egg rafts laid by the 0 dpe group, 27.5% were laid on the first day, and significantly more egg rafts were laid on the second day (72.5%) compared to the first day (*p* < 0.0001). In contrast, the 3 dpe group laid the largest proportion of eggs on the first day of oviposition (78.6%) and exhibited a more extended oviposition pattern, with egg deposition occurring from 1 to 4 days post-separation. On the second, third, and fifth days after join-up, significantly lower oviposition rates were observed in the 3 dpe group compared to the first day: 10.7% on day one (*p* < 0.0001), 3.6% on day three (*p* < 0.0001), and 7.1% on day five (*p* < 0.0001). The 5 dpe group produced a total of 11 egg rafts, distributed over the first (45.5%), second (27.3%), and fourth (27.3%) days. Significantly, fewer egg rafts were laid on the second day compared to the 0 dpe group (*p* < 0.0001).

Similar to the timing of oviposition onset, the oviposition pattern did not significantly vary between the different mating groups (0, 3, and 5 dpe, two-way ANOVA) for *Cq* (Figure 4B). In the 0 dpe group, oviposition began on the second day after separation, with from one to five egg rafts being laid daily until the eighth day, totaling 21 egg rafts. The 3 dpe group exhibited a 76.2% increase in the total number of egg rafts laid, as described above. However, high variance between experiments in these groups prevented the detection of a statistical difference. Oviposition in the 3 dpe group began on day 3 and continued daily except on day 6, resulting in a total of 37 egg rafts. The 5 dpe group showed a shortened oviposition period, with egg raft deposition starting on the third day and ending on the sixth day, totaling 30 egg rafts. These findings suggest that, while the oviposition patterns of *Cq* change with an increasing time to mating after emergence, these variations are not statistically significant.

### 3.3. Effect of Blood Compensation After Delayed Mating on Autogenous Culex pipiens Biotype Molestus Mosquitoes

**Figure 5:**
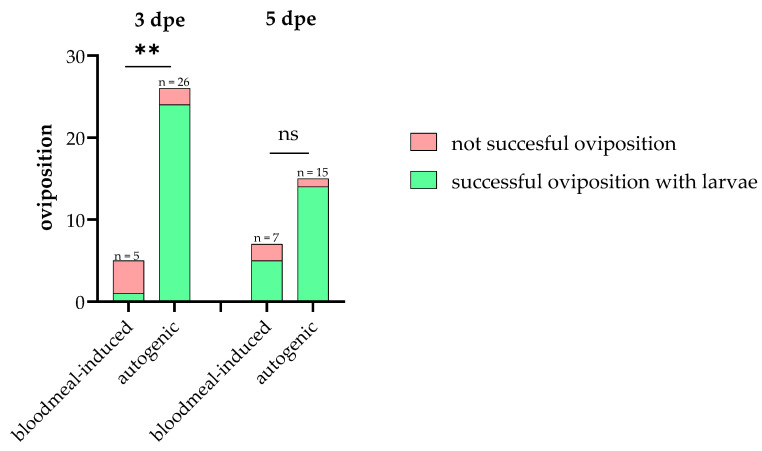
Comparison of autogenous and blood meal-induced oviposition between mated females 3 days (**left**) and 5 days (**right**) after emergence (dpe). Green: successful oviposition = egg rafts from which larvae hatched; red: unsuccessful oviposition = egg rafts from which no larvae hatched. Three days post-emergence: *p* = 0.0022 (**), autogenous oviposition significantly more successful; 5 dpe: *p* = 0.1404, no significant differences between periods. The bars represent the percentage of successful and unsuccessful ovipositions of three individual replicates for each group.

Since the decrease in egg production observed in the 3 and 5 dpe *Cm* mating groups could be attributed to resource depletion over the extended time period, we sought to determine whether a blood meal could restore egg production. Interestingly, the results reveal the opposite effect, showing that autogenous oviposition is significantly more efficient in terms of larval hatching. A statistically significant difference was observed between the autogenously and blood meal-induced successful ovipositions in the 3 dpe group (*p* = 0.0022; Fisher’s exact test), indicating that autogenous oviposition leads to higher larval hatching efficiency compared to blood meal-induced oviposition. Autogenous oviposition led to larval hatching in 92.3% of cases, significantly outperforming blood meal-induced oviposition, which achieved a larvae hatch rate of only 20%. Among all successful ovipositions (with larval hatching), 96% were contributed autogenously, with only 4% being blood meal-induced (Figure 5). The odds ratio was calculated as 0.02083, with a reciprocal odds ratio of 48 (95% CI: 0.001681–0.2898). This indicates that autogenous oviposition was approximately 48 times more likely to result in larval hatching compared to blood meal-induced oviposition. For the 5 dpe group, no statistically significant difference between the two groups was observed (*p* = 0.1404; Fisher’s exact test). However, the descriptive trends in the data suggest that autogenous oviposition may have an advantage in reproductive success under these conditions, as the reciprocal odds ratio was calculated as 9.33 (95% CI: 0.755–142.1), indicating a nine-times higher likelihood of successful autogenous oviposition (Table 3, Figure 5).

### 3.4. Protein Measurement

The current knowledge on oviposition in mosquitoes suggests that proteins are essential for egg development [30]. Therefore, the reduced oviposition observed in the 3 and 5 dpe mating groups may be due to the low protein content in the females. To investigate this, we quantified the protein content of *Cm* and *Cq* females at three time points post-emergence: immediately after emergence (0 dpe), 3 dpe, and 5 dpe (Supplementary Table S5, Table 4). The measurements were only taken from females that had not yet had a blood meal to exclude the effect of blood protein on our measurements. In the case of *Cq*, which is an anautogenic species, the average protein content per female was found to be 61.6 µg/mL at 0 dpe. In comparison, the *Cm* females exhibited an average protein content of 97.4 µg/mL at the same time point. In general, the protein measurement increased over time. For *Cq*, the protein value increased to 87 µg/mL per female in the 5 dpe group. In *Cm*, a value of 265.8 µg/mL per female was observed at 3 dpe, followed by a slight decline to 244.2 µg/mL per female in the 5 dpe group. Despite the gradual elevation in the protein content of the *Cq* females over time, it remained consistently lower than that of the *Cm* females in direct comparison.

## 4. Discussion

Despite decades of research into the factors influencing mosquito reproductive success, significant gaps persist in our understanding of several key aspects. Recent advancements have shed light on variables such as blood meal size, mosquito strains, host and breeding site availability, and mosquito density, which are critical determinants of reproductive outcomes [31,32,33]. For example, *Orthoflavivirus zikaense* (Zika virus, Orthoflavivirus) was shown to reduce the life span of *Ae. aegypti* females, but did not affect their oviposition [34]. In contrast, *Orthoflavivirus denguei* (DENV-2, Orthoflavivirus) infection of the same species reduced their life span and fecundity [35]. Furthermore, WNV infection led to a reduced fecundity of *Culex tarsalis* females but had no effect on their survival [36]. These differential effects of infection with arboviruses belonging to the same virus genus highlight the complex dynamics of virus–host interactions. In addition to the above-described effects of viral pathogens on the survival and overall fecundity of mosquitoes, the infection of *Cx. pipiens* with the protozoan parasite *Plasmodium relictum* resulted in earlier oviposition compared to uninfected individuals, demonstrating another possible effect of pathogen infection on the host [37].

In previous studies, we demonstrated that infection with the ISV CYV has no significant impact on the fecundity of *Cx. pipiens* biotype *molestus* mosquitoes compared to a control group (medium injected), provided the mosquitoes mated immediately after they were infected. In the same study, organ-specific analysis following CYV injection revealed rapid infiltration of the virus into the ovaries [13]. These results prompted us to investigate whether the timing of the first mating after CYV infection, following a defined incubation and replication period of the virus (i.e., 3–5 days post infection), could impact the reproductive outcomes. Our results indicate that this is not the case for either the autogenous species *Cx. pipiens* biotype *molestus* or the anautogenous subspecies *Cx. pipiens quinquefasciatus*. Even with extended incubation before mating, CYV infection did not result in any significant declines in key reproductive parameters such as the oviposition rates, larval hatch rates, or female mortality. We observed a decrease in the larval hatch rate in the 5 dpi CYV group compared to the medium-injected group. However, due to the high variability in the data, this difference was not statistically significant, and we cannot conclusively attribute it to CYV infection. Thus, CYV infection prior to mating does not appear to destabilize a naïve mosquito population. Not all individuals reached the detection limit of our CYV standard for virus quantification. In *Cx. pipiens quinquefasciatus*, those that fell below this threshold were 17–21 days old at the time of RT-qPCR. Since no long-term studies on CYV infection exist for this species, it remains uncertain whether the viral loads at 17–21 days post-infection were still high enough to be detected using our standard. Similarly, some individuals of *Cx. pipiens* biotype *molestus* did not meet the detection threshold and were excluded from the analyses. While it is possible that additional individuals in the reproductive success experiments also had undetectable viral loads, we nonetheless observed distinct differences between the two species. It should also be noted that the viral load differed between individuals. This is partly because they were infected for different lengths of time at the time of RT-qPCR, and partly because the initial infection may have had different efficacies depending on the replicate and virus aliquot. Nevertheless, we see clear differences between the different species. Interestingly, the timing of mating after emergence (0 dpe vs. 3 dpe and 5 dpe), regardless of infection status, had a significant impact on the reproductive parameters in *Cx. pipiens* biotype *molestus* but not in *Cx. pipiens quinquefasciatus*. In particular, the oviposition rate and reproductive success, defined as the proportion of females producing viable larvae relative to the total number of females tested, showed a decline in *Cx. pipiens* biotype *molestus*. This suggests that delayed mating reduces the reproductive success of *Cx. pipiens* biotype *molestus*.

The effect of reduced reproductive success was also detected in the second set of experiments without infection/injection, indicating that wounding of the female was not responsible for the observed effects. For the anautogenous species *Cx. pipiens quinquefasciatus*, a significant increase in reproductive success from day 0 (46.7%) to day 3 (84.1%) was detected, with a decline being observed on 5 dpe, although this change was not statistically significant. This is consistent with our first set of experiments using infected individuals but also with other studies investigating the relationship between age and fecundity, which have observed a continuous decline in fecundity starting from day 5 post-emergence [19].

In contrast to *Cx. pipiens quinquefasciatus*, *Cx*. *pipiens* biotype *molestus* successfully laid eggs at 0 dpe (88.9%), with a steady and significant decline in oviposition rates as mating was delayed. This brief interval between emergence and oviposition has been previously reported in a study of the same species that was conducted in Shanghai, China [38]. The authors hypothesized that the females of *Cx. pipiens* biotype *molestus* may be sexually receptive due to the early maturation of the ovaries shortly after emergence. Similar outcomes have been observed in *Cx. pipiens* biotype *molestus* strains from Australia, where the relatively short interval between emergence and initial oviposition is interpreted as an indicator of autogenesis [39]. This aligns with our findings on the high reproductive success rates of females mated immediately after emergence. By 3 dpe, only 64.4% of females laid eggs (compared to 5 dpe, *p* < 0.05), and a further significant decrease was observed at 5 dpe, with only 24.4% of females laying eggs (compared to 0 dpe, *p* < 0.01). When considering reproductive success, which encompasses additional factors beyond oviposition, a similar pattern emerged: at 5 dpe, fewer offspring were produced per female compared to at 0 dpe (*p* < 0.01), with a trend towards fewer than at 3 dpe (*p* > 0.05). The gonotrophic cycle, i.e., the process from ovarian development to oviposition, appears to be age-dependent in this species. In addition to the onset of ovipostion, we also assed the egg-laying patterns across different age groups for both mosquito species. The *Cx. pipiens* biotype *molestus* mosquitoes laid the majority of their eggs within 24 h over all three groups, with the 3 and 5 dpe groups continuing to lay eggs up to 5 days post-separation, suggesting a slight delay, although this was not statistically significant. In contrast, oviposition in the *Cx. pipiens quinquefasciatus* mosquitoes began, at the earliest, 48 h after separation into oviposition tubes in the 0 dpe group and after 72 h in the 3 dpe and 5 dpe groups. Notably, younger mated females were able to lay eggs over a longer duration, but with an overall lower egg raft output, while older females completed oviposition within a shorter timeframe. The highest oviposition rates were observed on day 3 in the 3 dpe group, with 62.2% of egg rafts being laid after separation into oviposition tubes, and day 4 in the 5 dpe group, with 46.7% of all laid eggs in this group. A gonotrophic cycle duration of from two to three days was described for *Cx. pipiens quinquefasciatus* [40]. Our results showed that this duration might vary with increasing age. In addition to age, the time of feeding and the provision of sugar water are also important, as described for *Cx. pipiens fatigans* (=*Cx. pipiens quinquefasciatus*). Studies have demonstrated that oviposition occurs in two distinct peaks that correspond to changes in light during the day: sunrise and sunset. These peaks influence the gonotrophic cycle and, consequently, the timing of oviposition [41,42]. It should also be noted that only blood-fed females were used in our experiments, but no assessment of mating success was made. Therefore, the lower reproductive success of females exposed to males immediately after emergence may be due to omitted mating rather than physiological reasons. The differences in the time span until oviposition between the species can be caused by various factors. It has already been described that female *Cx. pipiens* can delay their oviposition until finding an appropriate oviposition site but need to mate early after emergence [43]. This is consistent with our findings that the females of the autogenous species that were mated early had the highest reproductive success compared to those that were mated later. Overall, our results are supported by a study by Lea and Evans, in which they described that *Cx. pipiens* biotype *molestus* females were fertilized within 6 h of emergence, whereas *Cx. pipiens quinquefasciatus* females were at least 24 h old at the time of insemination [22]. Delayed mating in *Ae. aegypti* resulted in a 38% reduction in fecundity when they were mated eight days after emergence; however, the highest fecundity was measured when they were mated within the first two days after emergence [44]. The decline in fecundity from day five in *Ae. aegypti* is consistent with our results for the autogenous species, but different from the anautogenous species that we tested, suggesting that the reason for the differences we measured may not depend solely on autogeny versus anautogeny.

To add to this point, we analyzed if a blood meal could restore the egg-laying capacity of *Cx. pipiens* biotype *molestus* after delayed mating. This would support the hypothesis that older mosquitoes may suffer from nutrient and, in particular, protein deficiency, which could have prevented the production of viable egg rafts. As an autogenous species, *Cx. pipiens* biotype *molestus* females can typically produce eggs without requiring a blood meal, instead relying on stored nutrients accumulated during earlier developmental stages [45,46]. Autogenous oviposition is advantageous in environments where the presence of a host is intermittent, as well as in nutrient-rich environments. Our findings showed that most egg rafts were laid during the autogenous (non-blood-fed) phase, with the majority successfully developing into larvae. In contrast, the number of egg rafts significantly decreased after a blood meal, and for the 3 dpe group, the hatching rate was also significantly reduced. These results suggest that older mosquitoes, although still capable of laying eggs under certain conditions, may experience a decline in reproductive success due to age-related nutrient deficiencies, which a blood meal alone cannot fully compensate for.

Blood meals play a crucial role in the reproductive physiology of mosquitoes, not only by supporting egg development but also by serving additional functions. In some species, blood meals can enhance female nutrition, help sustain already-formed eggs, or be partially digested and excreted. For example, in *Ae. aegypti*, prolonged periods of starvation can increase the likelihood that gravid females will seek out an additional blood meal [47]. Notably, *Cx. pipiens* females are capable of retaining viable eggs for extended periods without the need for further blood consumption [48]. This ability highlights the complex interplay between blood meals and other nutritional sources in sustaining reproductive success across different mosquito species.

In a final experiment, the amount of protein was analyzed in both species at 0, 3, and 5 days after emergence. Proteins are important for embryogenesis after emergence in autogenous mosquitoes and are already stored at the larval stage [45,49,50]. We found that the amount of protein in autogenous *Cx. pipiens* biotype *molestus* females immediately after emergence is significantly lower than that in older females, which may be due to protein consumption during metamorphosis [50]. It has been described that the nutrient content of the fat body transferred from the larval to the adult stage is greater in autogenous than in anautogenous mosquitoes [49,51]. In relation to our measurements, a higher protein content can be seen in the autogenous species compared to the anautogenous species in all age groups that were measured. In anautogenous species, the synthesis of vitellogenins (yolk proteins) is also important for egg production [52]. However, this synthesis is only initiated by the ingestion of a blood meal through the release of corresponding hormones [53,54,55]. Although the amount of protein measured in the anautogenic *Cx. pipiens quinquefasciatus* females in our experiment was significantly lower, it also increased slightly over time. This increase in protein can only be due to protein synthesis, since the only food source was 8% fructose water. A comparative analysis of *Ae. aegypti* (autogenous) and *Aedes atropalpus* (autogenous) also revealed the presence of higher protein reserves in the autogenous species[56]. The authors argue that higher nutrient levels could stimulate the release of ovarian ecdysteroidogenic hormone (OEH) and insulin-like peptide 3 (ILP3), which are necessary for egg production and are triggered by blood meals in *Ae. aegypti*. Our data demonstrated a significant increase in protein content, particularly in the *Cx. pipiens* biotype *molestus* females that were examined 3 days and 5 days post-emergence and that were virgin. On day 3, the protein content in the female specimens was found to be approximately 2.7 times higher than the content observed on day 0. On day 5, the protein content in the female specimens was found to be approximately 2.5 times higher than the content observed on day 0. However, studies with *Anopheles gambiae* and *Drosophila melanogaster* have shown that the protein content decreases with age [57,58]. While protein levels increased in the older females in our experiments, the reproductive success decreased with age. This decline in reproductive success is not easily explained by a nutrient deficiency. Following emergence, a portion of the stored proteins is allocated toward vitellogenesis, resulting in a subsequent depletion of these reserves [50]. The fate of these proteins in the absence of mating and a suitable oviposition site remains to be elucidated. Besides nutrition availability, a further potential explanation for the deceasing reproductive success might be the influence of semen transfer on female behavior and physiology, as suggested by Villareal et al. (2018) [59]. The male accessory gland substances in *Ae. aegypti* have been demonstrated to exert a significant influence on female behavior with regard to blood feeding, fecundity, egg production, and survival [59].

Overall, this study has certain limitations. Not all individuals tested positive for CYV infection, highlighting the need for a more consistent time point for CYV RNA analysis in future experiments. Additionally, assessing the fertilization status of females would help to determine whether reduced reproductive success at later post-mating stages is due to unsuccessful mating or underlying physiological factors.

## 5. Conclusions

Overall, our study demonstrates that CYV infection in *Cx. pipiens quinquefasciatus* and *Cx. pipiens* biotype *molestus* mosquitoes did not result in significant changes to the reproductive parameters that were examined, even with an incubation period of up to 5 days. We also observed an age-dependent effect on the reproductive success of *Cx. pipiens* biotype *molestus*, while this was not evident in *Cx. pipiens quinquefasciatus*. Additionally, the reproductive decline in *Cx. pipiens* biotype *molestus* mosquitoes was not linked to protein deficiency, as compensatory blood meals did not improve the reproductive success and the protein levels did not decline with aging. These findings highlight the complexity of vector control strategies, suggesting that targeting the reproductive potential of mosquitoes may need to account for factors like age and nutritional status, rather than focusing solely on virus-induced effects. This approach will be essential in developing more effective methods for limiting mosquito populations and reducing the transmission of arboviruses.

## Figures and Tables

**Table 1:** Overview of the effects of CYV infection on mosquitoes’ reproductive success including oviposition rate, larval hatch rate, blood meal uptake, female mortality, and reproductive success of *Cx. pipiens quinquefasciatus* (*Cq*) and *Cx. pipiens* biotype *molestus* (*Cm*) at different days post-injection (dpi) with medium or CYV treatment. Mean data of at least two individual replicates are presented as percentages with raw counts in parentheses.

Species	dpi	Treatment	Oviposition Rate (Egg Raft/Total Females)	Larval Hatch Rate (Larval Hatch/Egg Rafts)	Female Mortality Rate (Dead/Total Females)	Blood Meal Uptake Rate (Engorged Female/Total Females)	Reproductive Success (Larval Hatch/Total Female)
*Cm*	0	Medium	88.9% (40/45)	100% (40/40)	0% (0/45)	/	88.9% (40/45)
		CYV	88.9% (40/45)	95% (38/40)	0% (0/45)	/	84.4% (38/45)
	3	Medium	46.7% (21/45)	81% (17/21)	0% (0/45)	/	37.8% (17/45)
		CYV	35.6% (16/45)	87.5% (14/16)	15.6% (7/45)	/	31.1% (14/45)
	5	Medium	54.8% (23/45)	78.3% (18/23)	0% (0/45)	/	40% (18/45)
		CYV	44.1% (21/45)	52.4% (11/21)	8.9% (4/45)	/	24.4% (11/45)
*Cq*	0	Medium	40% (18/45)	72.2% (13/18)	28.9% (13/45)	100% (45/45)	28.9% (13/45)
		CYV	52.3% (23/44)	69.6% (16/23)	22.7% (10/44)	100% (44/44)	36.4% (16/44)
	3	Medium	60.4% (32/53)	84.4% (27/32)	13.2% (7/53)	100% (53/53)	50.9% (27/53)
		CYV	62.9% (44/70)	75% (33/44)	18.6% (13/70)	100% (70/70)	47.1% (33/79)
	5	Medium	88.8% (71/79)	95.8% (68/71)	13.8% (11/80)	98.8% (79/80)	85% (68/79)
		CYV	85.4% (41/46)	95.1% (39/41)	20.8% (10/48)	95.8% (46/48)	81.3% (39/46)

**Table 2:** Overview of the effects of delayed mating on mosquitoes’ reproductive success including oviposition rate, larval hatch rate, blood meal uptake, female mortality, and reproductive success of *Cx. pipiens quinquefasciatus* (*Cq*) and *Cx. pipiens* biotype *molestus* (*Cm*) at different days post-emergence (dpe). Mean data of at least three individual replicates are presented as percentages with raw counts in parentheses.

Join up at dpe	Species (n in Total)	Oviposition Rate (Egg Raft/Total Females)	Larval Hatch Rate (Larval Hatch/Egg Rafts)	Female Mortality (Dead/Total Females)	Blood Meal Uptake Rate (Engorged Females/Total Females)	Reproductive Success (Larval Hatch/Total Female)
0	*Cm* (45)	88.9% (40/45)	100% (40/40)	2.2% (1/45)	/	88.9% (40/45)
	*Cq* (45)	46.7% (21/45)	66.7% (14/21)	6.5% (3/46)	97.8% (45/46)	31.1% (14/45)
3	*Cm* (45)	64.4% (29/45)	89.7% (26/29)	13.3% (6/45)	/	57.8% (26/45)
	*Cq* (44)	84.1% (37/44)	97.3% (36/37)	0% (0/46)	95.7% (44/46)	81.8% (36/44)
5	*Cm* (45)	24.4% (11/45)	90.9% (10/11)	8.9% (4/45)	/	22.2% (10/45)
	*Cq* (40)	75% (30/40)	100% (30/30)	2.2% (1/45)	88.9% (40/45)	75% (30/40)

**Table 3:** General distribution of oviposition and resulting successful ovipositions (with hatched larvae) in autogenous and blood meal-induced periods 3 and 5 days after emergence. Mean data of at least three individual replicates are presented as percentages, with raw values in parentheses.

		3 dpe	5 dpe
*Autogenous* *period*	Oviposition	52% (26/50)	36.6% (15/41)
	Successful oviposition	92.3% (24/26)	93.3% (14/15)
	Blood meal	87.5% (21/24)	84.6% (22/26)
*Post-blood feeding period*	Oviposition	23.8% (5/21)	22.7% (5/22)
	Successful oviposition	20% (1/5)	60% (3/5)

**Table 4:** Mean protein concentration (µg/mL) in *Cm* and *Cq* females at different days after emergence: 0, 3 and 5 dpe.

Species	Days Post Emergence [dpe]	Mean Protein Concentration Per Animal [µg/mL]
*Cm*	0	97.4
	3	265.8
	5	244.2
*Cq*	0	61.1
	3	75.1
	5	87

## Data Availability

The original contributions presented in this study are included in the article/supplementary material. Further inquiries can be directed to the corresponding author.

## Supplementary Materials

The following supporting information can be downloaded at: https://www.mdpi.com/article/10.3390/insects16030252/s1, Table S1: Raw data oviposition assay_CYV infection; Table S2: qPCR CYV quantification; Table S3: CYV effect; Table S4: Raw data oviposition assay_Cm vs. Cq; Table S5: BCA_Raw data
